# Review of the effects of vitexin in oxidative stress‐related diseases

**DOI:** 10.1002/fsn3.1567

**Published:** 2020-04-14

**Authors:** Fatemeh Babaei, Armita Moafizad, Zahra Darvishvand, Mohammadreza Mirzababaei, Hossein Hosseinzadeh, Marjan Nassiri‐Asl

**Affiliations:** ^1^ Department of Clinical Biochemistry School of Medicine Student Research Committee Shahid Beheshti University of Medical Sciences Tehran Iran; ^2^ Qazvin University of Medical Sciences Qazvin Iran; ^3^ Department of Clinical Biochemistry School of Medicine Kermanshah University of Medical Sciences Kermanshah Iran; ^4^ Pharmaceutical Research Center Pharmaceutical Technology Institute Mashhad University of Medical Sciences Mashhad Iran; ^5^ Department of Pharmacodynamic and Toxicology School of Pharmacy Pharmaceutical Technology Institute Mashhad University of Medical Sciences Mashhad Iran; ^6^ Department of Pharmacology and Neurobiology Research Center School of Medicine Shahid Beheshti University of Medical Sciences Tehran Iran

**Keywords:** antioxidant, lipid peroxidation, oxidative stress, reactive oxygen species, vitexin

## Abstract

Vitexin is an apigenin flavone glycoside found in food and medicinal plants. It has a variety of pharmacological effects, including antioxidant, anti‐inflammatory, anticancer, antinociceptive, and neuroprotective effects. This review study summarizes all the protective effects of vitexin as an antioxidant against reactive oxygen species, lipid peroxidation, and other oxidative damages in a variety of oxidative stress‐related diseases, including seizure, memory impairment, cerebral ischemia, neurotoxicity, myocardial and respiratory injury, and metabolic dysfunction, with possible molecular and cellular mechanisms. This review describes any activation or inhibition of the signaling pathways that depend on the antioxidant activity of vitexin. More basic research is needed on the antioxidative effects of vitexin in vivo, and carrying out clinical trials for the treatment of oxidative stress‐related diseases is also recommended.

## Graphical abstract

Vitexin is an apigenin flavone glycoside found in food and medicinal plants. Vitexin is an antioxidant against reactive oxygen species and lipid peroxidation. Vitexin activates the signaling pathways that depend on its antioxidant activity.

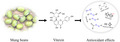

## 1 INTRODUCTION

Vitexin (Apigenin‐8‐C‐β‐D‐glucopyranoside) is a chemical compound found in many plants, such as buckwheat (Zielinska, Szawara‐Nowak, Ornatowska, & Wiczkowski, 2007), hawthorn (Kirakosyan et al., 2003), Echinodorus (Tanus‐Rangel et al., 2010), bamboo (Wang, Yue, Jiang, & Tang, 2012), mung bean (Cao et al., 2011), and Passiflora (Gadioli, da Cunha, de Carvalho, Costa, & Pineli, 2018). Vitexin is found as a major polyphenol in food sources such as mung beans (Hou et al., 2019).

Mung bean is consumed as soup and is a popular food item in China and many Asian countries, where it is believed to control heatstroke (Cao et al., 2011). Vitexin has a variety of pharmacological effects, including antioxidant (Bai et al., 2016), anti‐inflammatory (Choi et al., 2014; Nikfarjam, Hajiali, Adineh, & Nassiri‐Asl, 2017), anticancer (Yang et al., 2013), anticholinesterase (Sheeja Malar, Beema Shafreen, Karutha Pandian, & Pandima Devi, 2017), antibacterial (Quílez et al., 2010; Das et al., 2016), antiviral (Fahmy et al., 2020), antinociceptive (Borghi et al., 2013), hepatoprotective (Kim, Chin, Lim, Kim, & Kim, 2004), cardioprotective (Dong et al., 2013), and neuroprotective effects (Yang, Yang, Zhang, Tian, Liu, & Zhao, 2014; Hosseinzadeh & Nassiri‐Asl, 2017).

**FIGURE 1:**
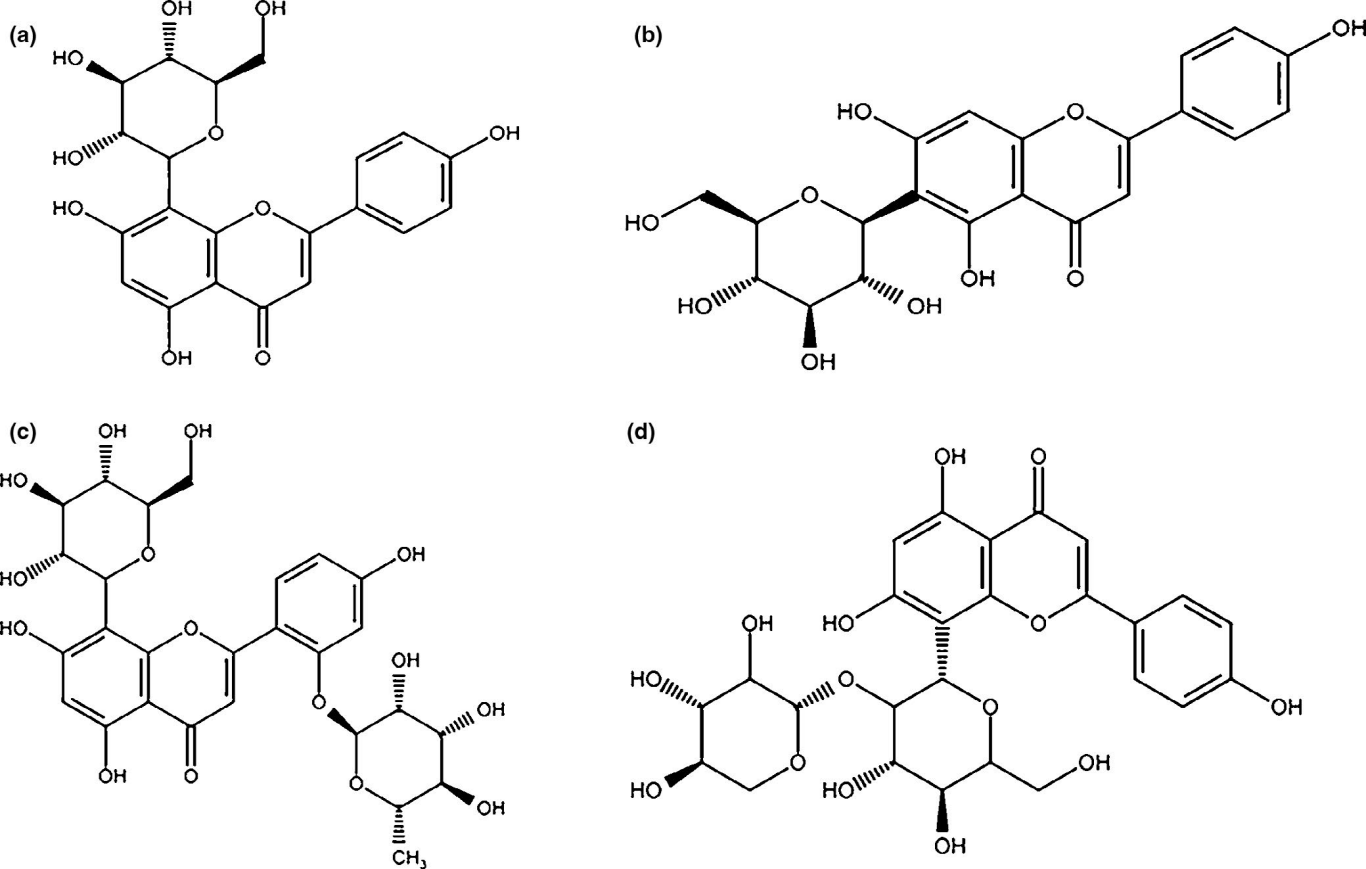
Chemical structures of vitexin and some derivatives. (A) Vitexin, (B) isovitexin, (C) vitexin‐2‐O‐rhamnoside, and (D) vitexin‐2‐O‐xyloside

Vitexin has been proven capable of donating electrons and has acted as a good radical scavenger. It has a better antioxidant activity than apigenin, since the presence of C‐8 glucoside in vitexin causes a reduction of its bond dissociation enthalpy compared to aglycone apigenin. The most stable radical order of vitexin after reaction with reactive oxygen species (ROS) was reported as 4′‐OH, 7‐OH, and 5‐OH, respectively (Praveena, Sadasivam, Kumaresan, Deepha, & Sivakumar, 2013). Vitexin has some derivatives too, such as isovitexin, rhamnopyranosyl‐vitexin, methylvitexin (isoembigenin), vitexin‐2‐O‐rhamnoside (VOR), and vitexin‐2‐O‐xyloside (VOX; Figure 1; Ninfali, Antonini, Frati, & Scarpa, 2017; Praveena et al., 2013).

Vitexin is poorly absorbed in the gastrointestinal tract. It is rapidly removed from the blood, and its absolute oral bioavailability is very low. Vitexin is probably deglycosylated as the first step and converted to 3‐(4‐hydroxyphenyl) propionic acid in the end. The first‐pass effects of vitexin are almost intestinal (approximately 94%) and less gastric (30%) and hepatic (5%), which contribute to its low bioavailability. Vitexin is rapidly and widely distributed into various tissues. Vitexin is excreted most in the urine and bile (Ninfali & Angelino, 2013; Xue et al., 2014).

**FIGURE 2:**
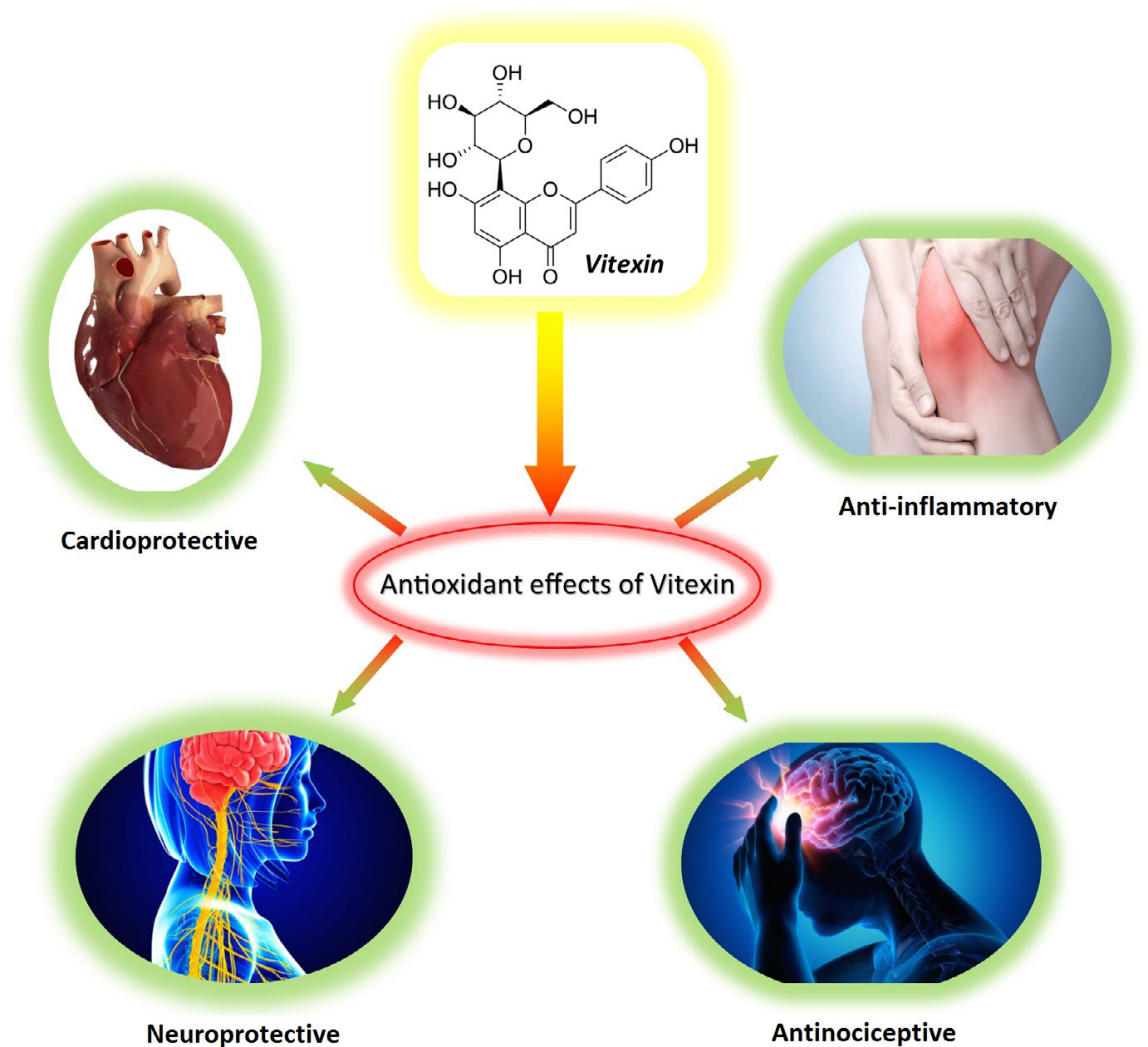
Antioxidative effects of vitexin in oxidative stress‐related diseases [Correction added on 24 April 2020, after first online publication: Figure 2 has been corrected.]

Recently, the nanoparticles of vitexin have increased its rate of dissolution despite the low aqueous solubility of the raw drug (Gu et al., 2017). In recent years, an increasing attention has been paid to the search for natural antioxidants, and vitexin has received great attention due to its antioxidant activities. This review study thus summarizes the antioxidant effects of vitexin and its derivatives on oxidative stress‐related diseases (Figure 2).

## 2 METHODS

All the major in vivo or in vitro studies conducted over the past decade about the effects of vitexin as an antioxidant on oxidative stress were selected for this review study. All the studies related to herbal medicines in which vitexin plays a major role as an antioxidant were also selected. Scopus, PubMed, and Web of Science were used as the databases, and the search was focused on the effect of vitexin on oxidative markers, antioxidant enzymes, and any signaling and gene expression potentially involved in its protective effects. The keywords used for the search were as follows: vitexin, vitexin and antioxidant, vitexin and oxidative stress.

## 3 OXIDATIVE STRESS‐RELATED DISEASES

### 3.1 Neurological and psychiatric disorders

Vitexin (10 mg/kg, p.o., 16 days) has antiepileptic effects on pilocarpine (85 mg/kg) model. Vitexin attenuated the increment of lipid peroxidation and the nitrite content and neural loss and restored acetylcholinesterase–monoamine oxidase to the normal levels. It also reduced the mRNA expression of N‐methyl‐D‐aspartate receptor (NMDAR), metabotropic glutamate receptor 1 (mGluR1), and metabotropic glutamate 5 (mGlu5) receptor (Aseervatham, Suryakala, Doulethunisha Sundaram, Bose, & Sivasudha, 2016).

Vitexin compound B‐1 (10^–7^ and 10^–6^ M) showed dose‐dependent neuroprotective effects against hypoxia/reoxygenation‐induced oxidative injury in PC‐12 by reducing caspase 3/7‐like activities, ROS production, 4‐hydroxynonenal and malondialdehyde (MDA) levels and NADPH oxidase‐2 (NOX2) and NOX4 expression (Yang, Tan, et al., 2014).

Vitexin (15 mg/kg, i.v.) ameliorated neurological defects in cerebral ischemia/reperfusion (I/R) by increasing the extracellular signal‐regulated kinases1/2 (p‐ERK1/2) and the Bcl‐2 protein level in the cortex and hippocampus and attenuating the level of c‐Jun N‐terminal kinases3 (p‐JNK), p38 phosphorylation, and Bax expression (Wang et al., 2015).

Pretreatment with vitexin (2 mg/kg, i.v.) suppressed the apoptosis induced by middle cerebral artery occlusion (MCAO) by decreasing the secretion of pro‐inflammatory cytokines (TNF‐α and IL‐6) and increasing anti‐inflammatory cytokines (IL‐10), and decreasing the expression of autophagy‐related proteins (mTOR, Ulk1, PPAR‐γ, Beclin1 p62, and LC3; Jiang, Dai, & Cui, 2018).

Vitexin (45 mg/kg, i.p.) showed significant neuroprotective effects following hypoxic/ischemic injury (HI) and reduced brain edema, neuronal cell death, the brain infarct volume, and blood–brain barrier (BBB) breakdown in rat pups. Vitexin inhibited the upregulation of hypoxia‐inducible factor (HIF)‐1α and vascular endothelial growth factor (VEGF) significantly. By inhibiting HIF‐1α, vitexin had long‐term neuroprotective effects in both morphology and neurological function after neonatal HI injury (Min et al., 2015).

Table 1 presents the effects of pretreatment with vitexin on glutamate toxicity. Pretreatment with vitexin (50 µM) demonstrated significant antioxidant and antiapoptotic effects on glutamate‐induced neurotoxicity in neuro‐2a cells. Vitexin also increased the clearance of glutamate by regulating glutamate transporters GLAST‐1 and GLT‐1 (Malar, Prasant, Shafreen, Balamurugan, & Devi, 2018; Table 1).

**TABLE 1:** Effect of vitexin on oxidative stress in some neurotoxicity models

Vitexin	Study	Oxidative and defense biomarkers	Signaling and gene expression	Ref.
In vitro concentration				
10 µM	NMDA (200 μM) and glycine (10 μM)‐induced toxicity in cultured cortical neurons		Increased Bcl‐2 Decreased Bax protein and the ratio of Bax/Bcl‐2 expression Downregulated the protein levels of NR2B‐containing NMDA receptors Reduced the overload of intracellular Ca^2+^	Yang, Yang, Zhang, Tian, Liu, and Zhao (2014)
10 and 100 µM, 24 hr	Exposure to isoflurane (1.4%) in human PC12 cells	Decreased ROS levels, increased GSH and SOD	Inhibited the level of pro‐inflammatory cytokines (TNF‐α and IL‐6) Decreased caspase‐3, BACE protein expression levels, cytosolic calcium levels, TRPV1, and NR2B protein expression levels	Chen, Zhang, Shan, and Zhao (2016)
50 µM	Glutamate (5 mM)‐induced cytotoxicity in Neuro‐2a cells	Decreased MDA and NO production	Upregulation of antioxidant response genes (Nrf2, HO‐1, NQO‐1, and Grp78) Downregulated Gadd153 Preserved MMP Suppressed cyclophilin D expression Downregulated NMDR and calpain gene expression Increased Bcl‐2/Bax ratio Decreased caspase‐3 Increased GLAST‐1, GLT‐1)	Malar, Prasant, et al. (2018)

Abbreviations: BACE, β‐site amyloid precursor protein (APP) cleaving enzyme 1; Gadd153, Growth arrest and DNA damage 153; GLAST‐1; GLT‐1, Glutamate transporters; Grp 78,78‐kDa Glucose‐regulated protein; GSH, Glutathione; Heme oxygenase 1; HO‐1; MDA, Malondialdehyde; MMP, Mitochondrial membrane potential; NMDA, N‐methyl‐D‐aspartate; NO, Nitric oxide; NQO‐1, NADH‐quinone oxidoreductase; NR2B, N‐methyl D‐aspartate receptor subtype 2B; Nrf‐2, Nuclear factor erythroid 2‐related factor 2; ROS, Reactive oxygen species; SOD, Superoxide dismutase.

Vitexin (10–40 μM) protected the dopaminergic neurons against methyl‐4‐phenylpyridinium (MPP^+^)‐induced toxicity and apoptosis and also decreased the expression of caspase‐3 and Bax/Bcl‐2 ratio in a dose‐dependent manner in the SH‐SY5Y cells. Vitexin (50 mg/kg) prevented bradykinesia and initial lesions caused by 1‐methyl‐4‐phenyl‐1,2,3,6‐tetrahydropyridine (MPTP) in Parkinson's disease in mice. In both in vitro and in vivo studies, vitexin was found to activate PI3K/akt signaling pathway (Hu, Li, & Wang, 2018).

Vitexin (10–30 mg/kg, i.p.) also reduced the immobility time in both the tail‐suspension test and the modified forced swimming test in mice, which is attributed to its antidepressant‐like effects. The antidepressant effects of vitexin may be related to increasing catecholamine in synaptic cleft, activating serotonergic 5‐HT_1A_, noradrenergic α_2_, and dopaminergic D_1_, D_2_, and D_3_ receptors (Can et al., 2013).

### 3.2 Memory impairment

Vitexin (150 µg/ml) as a glycosylated flavonoid isolated from *Serjania erecta* leaves, strongly protected the PC12 cells against Aβ_25‐35_ peptide‐induced toxicity when the cells were treated with it prior to Aβ_25‐35_ peptide. Vitexin inhibited amyloid β25‐35 peptide‐induced nitric oxide (NO) generation in PC12 cells, which explains the protective mechanism of it against Aβ25‐35 peptide‐induced toxicity (Guimarães et al., 2015).

Pretreatment with vitexin (50 μM) reduced oxidative stress and reactive nitrogen species (RNS) caused by the Aβ_25‐35_ peptide in a dose‐dependent manner. It also inhibited Aβ_25‐35_ peptide aggregation by interaction with Ile31, Gly33, and Met35 residues in the Aβ_25‐35_ peptide and by the interaction created among the peptides and hampering β‐sheet formation. Vitexin (50 μM) protected the neuro‐2a cells from Aβ25‐35 toxicity through the nuclear factor erythroid 2‐related factor 2/Heme oxygenase‐1 (Nrf‐2/HO‐1)‐dependent antioxidant pathway, modulated the genes involved in the antioxidant response pathway (such as ABCA1, ApoE, seladin‐1, Cyclophilin D (CypD)‐related gene, and unfolded protein response (UPR) specific genes), contributed to lipid metabolism, helped maintain the mitochondrial membrane potential, and inhibited the expression of apoptotic proteins (Malar, Suryanarayanan, et al., 2018).

Two flavonoids (vitexin and quercetin 3‐O‐glucoside), isolated from *Nelumbo nucifera* embryos, showed a potent inhibitory activity against β‐site amyloid precursor protein (APP) cleaving enzyme 1 (BACE1) and Cholinesterase (ChE). Vitexin also demonstrated more potent inhibitory activity against BACE1 and ChEs compared to quercetin 3‐O‐glucoside (Jung, Karki, Kim, & Choi, 2015).

Vitexin (100 µM) showed significant cholinesterase inhibitory effects for both acetylcholinesterase and butyrylcholinesterase activity (Sheeja Malar et al., 2017). As an antioxidant, vitexin (40 mg/kg) increased the total antioxidant capacity, superoxide dismutase, catalase, and glutathione peroxidase activities in the serum and also the levels of superoxide dismutase, catalase, glutathione peroxidase, Na^+^‐K^+^‐ATP enzyme, and Ca^2+^‐Mg^2+^‐ATP enzyme in the liver, brain, and kidneys in D‐galactose model of aging in mice. Vitexin also reduced MDA levels in the liver, brain, and kidney and lipofuscin levels in the brain too. In addition, the neuronal ultrastructure was improved by vitexin (An, Yang, Tian, & Wang, 2012).

Vitexin (100 μM) improved memory retrieval in scopolamine model of memory impairment in rats (Abbasi, Nassiri‐Asl, Sheikhi, & Shafiee, 2013). The modulatory effect of vitexin on cholinergic system was mentioned for possible mechanism, since it was shown that scopolamine causes rising in brain acetylcholinesterase enzyme (AChE) activity and brain oxidative stress (El‐Khadragy, Al‐Olayan, & Abdel Moneim, 2014).

Vitexin (3, 10 mg/kg) could reverse escape latency period in Morris water maze test against memory impairment of isoflurane in rats. Vitexin (10, 100 µM) could also increase cell viability of PC‐12 cells against neurotoxicity of isoflurane and reduce inflammatory cytokines (TNF‐α, Il‐6) and ROS and increase glutathione (GSH) and superoxide dismutase (SOD). Vitexin also reduced apoptosis in both PC‐12 cells and hippocampus neurons and increased expression mir‐409 in both models. Vitexin has protective effects against oxidative stress and inflammation induced by isoflurane and the underlying mechanism is probably through activation AMPK/GSK3β signaling pathway (Qi, Chen, Shan, Nie, & Wang, 2020).

**FIGURE 3:**
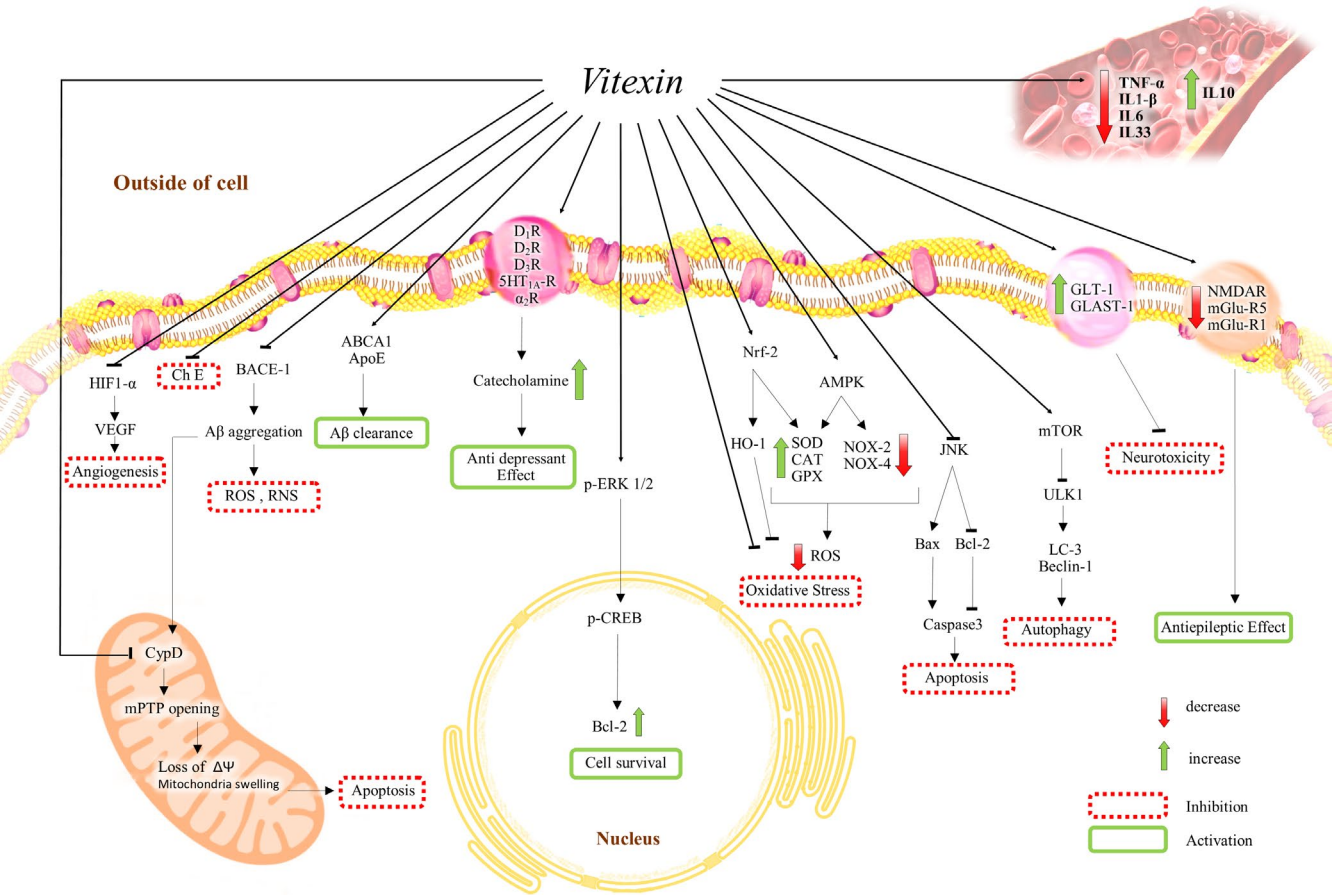
Possible signaling of vitexin against oxidative stress in different diseases in noncancerous cell. Aβ, β‐amyloid; ABCA‐1, ATP‐binding cassette transporter 1; AMPK, AMP‐activated protein kinase; ApoE, apolipoprotein E; α_2_R, α_2_ Adrenergic receptor; BACE1, β‐site amyloid precursor protein (APP) cleaving enzyme 1; ChE, Cholinesterase; CAT, Catalase; CypD, Cyclophilin D; D_1,2,3_ Rs, D_1,2,3_ receptors; GLAST‐1 and GLT‐1, Glutamate transporters; GPX, Glutathione Peroxidase; HIF‐1α, Hypoxia‐inducible factor 1; HO‐1, Heme oxygenase‐1; 5‐HT_1A_ R, 5‐HT_1A_ receptor; JNK, c‐Jun N‐terminal kinases3; mGluR1 and mGlu5, Metabotropic glutamate receptor 1 and 5; mPTP, Mitochondrial permeability transition pore; mTOR,Mammalian target of rapamycin; NMDAR, N‐methyl‐D‐aspartate receptor; p‐CREB, Phosphorylated cAMP response element‐binding protein; ROS, Reactive oxygen species; SOD, Superoxide Dismutase; RNS, Reactive nitrogen species; NOX2,4, NADPH oxidase‐2 and 4, Nrf‐2, Nuclear factor‐E2‐related factor 2; p‐ERK1/2, Extracellular signal‐regulated protein kinases 1 and 2; ULK1, Unc‐51 like autophagy activating kinase; VEGF, Vascular endothelial growth factor

Figure 3 presents a summary of the studied effects of vitexin against oxidative stress via different signaling pathways in cells. This figure shows the effects of vitexin on the membrane receptors and its role in the transporter system and how it activates Nrf‐2, AMPK, mTOR, and ABCA1 and inhibits HIF‐1α, BACE1, ChEs, JNK, and CypD in noncancer cells.

### 3.3 Antinociceptive and anti‐inflammatory activities

Vitexin (10 mg/kg, i.p., 30 min before stimulus with phenyl‐p‐benzoquinone, 1,890 μg/kg) inhibits inflammation‐associated pain and can also inhibit 91% of the acetic acid‐induced writhing response and pain‐like behavior induced by phenyl‐p‐benzoquinone, complete Freund's adjuvant, capsaicin (an agonist of transient receptor potential vanilloid 1, TRPV1), and both phases of the formalin test. As the possible mechanism, vitexin could prevent the reduction of glutathione levels, the ferric‐reducing ability potential, and the free‐radical scavenger ability, inhibit the production of hyperalgesic cytokines, such as TNF‐α, IL‐1β, IL‐6, and IL‐33, and upregulate anti‐hyperalgesic cytokine IL‐10 levels (Borghi et al., 2013). Figure 3 also shows the role of vitexin in the activity of peripheral cytokines in the peripheral system.

### 3.4 Cardiovascular injury

Vitexin preconditioning (100 µM, for 20 min, 24 hr) before anoxia and reoxygenation on cultured neonatal rat cardiomyocytes enhanced cell viability, creatine kinase (CK), and lactate dehydrogenase (LDH) as ischemic indexes by decreasing the apoptotic cells and intracellular Ca^2+^ overload and increasing extracellular signal‐regulated protein kinases (ERK1/2) activity in neonatal rat cardiomyocytes after anoxia‐reoxygenation (Dong, Chen, Guo, Cheng, & Shao, 2008).

Vitexin (6 mg/kg, i.v.) has cardioprotective effects and decreases the elevation of the ST segment of ECG and reduces myocardial infarct size in myocardial ischemia‐reperfusion in rats. It also reduced LDH and CK activities and MDA level and increased SOD in the serum. Vitexin decreased myocardial NF‐κB, TNF‐α, phosphorylated c‐Jun, and phosphorylated ERK expression in myocardial tissue (Dong et al., 2013).

Isoproterenol infusion and transverse aortic constriction increased ROS levels and induced cardiac hypertrophy in both in vitro and in vivo models. Vitexin (30 mg/kg, i.p., 100 μM) reduced hypertrophic markers such as atrial natriuretic peptide (ANP), brain natriuretic peptide (BNP), and β‐MHC at the mRNA and protein levels in both models. Vitexin (100 μM) also decreased the enhancement of intracellular calcium in isoproterenol‐induced cardiac hypertrophy in cultured neonatal rat myocytes. It also inhibited calcineurin–nuclear factor of activated T‐cells c3 (NFATc3) and phosphorylated calmodulin kinase II (CaMKII) in both models as calcium downstream effectors, which are involved in cardiac hypertrophy and heart failure (Lu et al., 2013). Some antioxidant effects of vitexin on oxidative stress in different models of cardiovascular injury are presented in Table 2.

**TABLE 2:** Antioxidant effects of vitexin on some oxidative stress models

Vitexin	Study	Oxidative markers and antioxidant enzymes	Signaling and gene expression	Ref.
In vitro concentration				
400 μM	H_2_O_2_ (180 µM)‐induced oxidative stress in HUVECs	Decreased ROS levels Inhibited LPO		Ugusman, Zakaria, Hui, Nordin, and Mahdy (2012)
Pretreatment (20 μM)	HUVECs treated with oxidized‐LDL	Reduced ROS and MDA levels Increased SOD activity	Increased the expression of p‐AMPK Decreased the expression of p‐mTOR	Zhang et al. (2017)
30 and 60 µg/ml	H_2_O_2_ (400 mM)‐induced oxidative damage in human erythrocytes	Reduced the erythrocyte hemolysis, formation of methemoglobin, skeleton protein damage, ROS, and MDA contents Enhanced the activities of SOD, CAT and GPx, and sulfhydryl content		An, Cao, Qu, and Wang (2015)
10 µM	H/R in H9c2 cells I/R injury in isolated rat heart	Reduced ROS levels	Decreased expression NOX4, inhibited the release of Cyt c from mitochondria into the cytoplasm, reduced cleaved caspase‐3/9 expression in both models Increased the Bcl‐2/Bax ratio in rat heart	Xue et al. (2020)
20 µM, 24 hr	Ethanol (100 µM)‐induced LO2 cell injury, 24 hr	Decreased TNF‐α, IL‐1β, IL‐6, and MDA levels	Increased the expression of Nrf‐2 and HO‐1 Inhibited the expression of NLRP3	Yuan et al. (2020)
In vivo dose				
60 mg/kg, i.p.	L‐NAME induced preeclampsia rat model	Decreased MDA level Increased SOD activity	Decreased expression of sFlt‐1, PlGF, TFPI‐2, HIF 1α, and VEGF	Zheng et al. (2019)
30 mg/kg, p.o.	Doxorubicin‐induced acute cardiotoxicity rat model	Reduced MDA, IL‐1β, IL‐6, NF‐κB, and TNF‐α levels Increased SOD, CAT, and myeloperoxidase activities	Reduced caspase‐3 activity Increased FOXO3a expression	Sun et al. (2016)
Post‐treatment (1.5 mg/kg, p.o.)	Isoproterenol‐induced heart damage in rats	Increased the levels of SOD, CAT, GPx, and nonenzymatic antioxidants vitamin C, E, and GSH Reduced the MDA level		Ashokkumar, Jamuna, Sakeena Sadullah, and Niranjali Devaraj (2018)
80 mg/kg, 4 weeks	Liver damage induced by ethanol (30%,40%,50%,55%, 4 weeks) in mice	Decreased MDA and TNF‐α levels and increased SOD	Increased expression of Sirt1 and Bcl‐2, inhibited apoptosis (Bax, ac‐p53, cleaved caspase‐3)	Yuan et al. (2020)

Abbreviations: ac‐p53, Acetylated p53; AMPK, AMP‐activated protein kinase; CAT, Catalase; Cyt c, Cytochrome c; FOXO3, Forkhead‐box protein O class subfamily 3; GPx, Glutathione peroxidase; GSH, Glutathione; H/R, Hypoxia/Reoxygenetion; H_2_O_2_, Hydrogen peroxide; HIF‐1α, hypoxia‐inducible factor 1; HO‐1, heme oxygenase 1; HUVECs, Human umbilical vein endothelial cells; I/R, Ischemia/Reperfusion; LDL, Low‐density lipoprotein; L‐NAME, N omega‐nitro‐L‐arginine methyl ester; LPO, Lipid peroxidation; MDA, Malondialdehyde; mTOR, mammalian target of rapamycin; NLRP3, NLR Family Pyrin Domain Containing 3; NOX4, NADPH oxidase 4 (NOX4);Nrf‐2, nuclear factor erythroid 2‐related factor 2; PlGF, Placental growth factor; ROS, Reactive oxygen species; sFlt‐1, soluble FMS‐like tyrosine kinase‐1; Sirt1, Silent information regulator 1; SOD, Superoxide dismutase; TFPI‐2, Tissue factor pathway inhibitor 2; VEGF, Tissue factor pathway inhibitor 2.

### 3.5 Respiratory injury

Vitexin (10 mg/kg, i.p.) suppressed LPS‐induced acute lung injury by increasing the expression of nuclear factor erythroid‐2‐related factor2 (Nrf2) and the activation of heme oxygenase (HO)‐1 in mice. Also, TNF‐α, IL‐1β, IL‐6, and MDA production were decreased by vitexin (Lu, Yu, Liu, & Gu, 2018). The Nrf2/HO‐1 pathway was found to have a potential protective role against oxidative stress (Nikam et al., 2016). The antioxidant effect of vitexin has been attributed to the activation of this pathway. Vitexin also inhibited NLR Family Pyrin Domain Containing 3 (NLRP3) expression. An interesting issue is that the noted effect of vitexin was removed in Nrf2−/− mice (Lu, Yu, Liu, & Gu, 2018). Furthermore, ROS, IL‐1β, IL‐6, TNF‐α, and MDA levels were decreased by vitexin (50 μM) in LPS‐activated RAW cells. Similarly, the knockdown of Nrf2 by siRNA in RAW cells suppressed the benefits of vitexin in an in vitro study. Figure 3 shows how vitexin activates Nrf‐2 and HO‐1 (Lu, Yu, Liu, & Gu, 2018).

### 3.6 Other antioxidative studies

Table 2 presents a summary of other studies conducted on the antioxidant effects of vitexin. In addition, there are several studies that have worked on the total extract of herbs that contain vitexin and have antioxidant activities due to vitexin. The present review study summarized some of the most important of these studies. Cardioprotective effects have been demonstrated for mung bean polyphenol extract on aluminum‐induced myocardial injury in rats. The major polyphenols of this extract include vitexin and isovitexin (Cheng, Wang, Wang, & Hou, 2017; Table 3).

**TABLE 3:** The effects of vitexin in herbal extract on oxidative markers and antioxidant enzymes

Herbal extract	Study	Oxidative markers and antioxidant enzymes	Signaling and gene expression	Ref
In vitro concentration				
Mung bean soup (30 g/1,000 ml)	DPPH, FRAP, ABTS	Higher ability of DPPH and ABTS˚^+^ radical scavenging, and increased FRAP		Li et al. (2012)
*Ficus deltoidea* leaves 50% ethanol–water extract (percentage yield: 25.2 ± 0.1%; Vitexin: 0.62 ± 0.01%)	DPPH	Highest DPPH, radical scavenging activity		Abu Bakar, Manaharan, Merican, and Mohamad (2018)
*Acer palmatum* ethanolic extract (Vitexin 50 μg/ml)	UVB‐irradiated HDFs	Reduced ROS production		Kim et al. (2005)
*Zanthoxylum bungeanum*leaves 95% ethanolic extract (1,824.4 g)	TBARS assay	Inhibited lipid peroxidation (Vitexin, IC_50_ = 0.014 ± 0.001 mM)		Zhang, Wang, Yang, Zhou, and Zhang (2014)
Ethyl acetate fraction (EAF) of *Nectandra cuspidata* leaves (Vitexin 2 µg/ml)	L‐929 fibroblasts irradiated with UVB (500 mJ/cm^2^)	Increased cell viability Inhibited the UVB‐induced ROS production and LPO		Ferreira et al. (2020)
In vivo dose				
Mung bean coat extract (400 mg/kg, gavage)	Heat stress in rats (swimming cells at 40 ± 1°C for 30 min)	Reduced the levels of MDA, LDH, and NOS, increased the levels of total antioxidant capacity and GSH		Cao et al. (2011)
Mung bean polyphenol extract 200 mg kg^−1^ day^−1^, 12 weeks	Myocardial injury by aluminum (171.8 mg/kg, 12 weeks) in rats	Reversed decrement of SOD, CAT, GPx, GST, and GSH Reversed increment of CK, LDH, MDA, GSSG, GSH, and AOPP Increased Na^+^/K^+^‐ATPase activity Reduced Ca^2+^‐ATPase activity, and Na^+^, Ca^2+^ ion levels	Inhibited ROS‐triggered Ca^2+^/JNK/NF‐κB signaling pathway, reduced caspase‐9 and cytochrome C expression	Cheng, Wang, Wang, and Hou (2017)
Dehydrated beet stalks and leaves 3.07 mg of vitexin‐rhamnoside equivalents 100 g^−1^, 8 weeks	High‐fat diet‐induced oxidative damage in liver in mice	Reversed increment of MDA level, GPx, and GR activities, improved total cholesterol level		Lorizola et al. (2018)
*F. carica* fruit extract (400 mg/kg, 8 weeks)	High‐fat diet (normal diet supplemented with 1% cholesterol, 4% fat, and 0.1% cholic acid)‐induced hyperlipidemic rats	Reduced the levels of plasma cholesterol, TG, LDL‐C, and AI, increased HDL‐C concentration, decreased TBARS, increased GPx, SOD, and CAT in liver, heart, and kidney		Belguith‐Hadriche et al. (2016)
Methanolic extract of *Ficus deltoidea* leaves (1 g/kg, gavage, 8 weeks) Vitexin (1 mg/kg, gavage, 8 weeks)	STZ‐induced diabetic rats	Extract increased both pancreatic GPx and SOD values Vitexin only increased GPx level Both reduced TBARS value		Nurdiana et al. (2017)
Methanolic extract of *Vigna angularis* Vitexin (50, 100 μM)	Thermal and oxidative stress in Caenorhabditis elegans	Reduced ROS levels, increased catalase and SOD activities	Upregulated SOD‐3 and HSP‐16.2 expressions in transgenic nematodes	Lee et al. (2015)

Abbreviations: ABTS, 2,2'‐azino‐bis‐(3‐ethylbenzothiazoline‐6‐sulphonic acid) diammonium salt; AI, Atherogenic index; AOPP, Advanced oxidation protein products; CAT, Catalase; CK, Creatine kinase; DPPH, 2,2‐Diphenyl‐1‐picrylhydrazyl; FRAP, Ferric reducing antioxidant power; GPx, Glutathione peroxidase; GR, Glutathione reductase; GSH, Glutathione; GSSG, Oxidized glutathione; GST, Glutathione S‐transferase; HDFs, Human dermal fibroblasts; HDL‐c, High‐density lipoprotein cholesterol; HSP, Heat shock protein; JNK/NF‐κB, c‐Jun N‐terminal kinase/nuclear factor‐kappaB; LDH, Lactate dehydrogenase; LDL‐c, Low‐density lipoprotein cholesterol; LPO, Lipid peroxidation; MDA, Malondialdehyde; NOS, Nitric oxide synthase; ROS, Reactive oxygen species; SOD, Superoxide dismutase; STZ, Streptozotocin; TBARS, Thiobarbituric acid reactive substances; TG, Triglyceride; UVB, Ultraviolet B.

Vitexin and isovitexin, as major antioxidant components in various cultivars of mung bean, may be involved in DPPH and ABTS ˚^+^ radicals’ scavenging abilities, and FRAP (ferric reducing antioxidant power) in MBS (Table 3). Nonetheless, this effect was greater in the MBS of cv. Huang and cv. Mao than cv. Ming (Li, Cao, Yi, Cao, & Jiang, 2012).

The methanolic extract of *Ficus deltoidea* leaves (1 g/kg) and vitexin (1 mg/kg) attenuated pancreatic oxidative damage and prevented β‐cell destruction in diabetic rats (Nurdiana et al., 2017; Table 3).

## 4 CANCER

As previously described, vitexin inhibits apoptosis in noncancerous cells and acts as antioxidant. On the other hand, it has different effect on apoptosis in tumor cells. Vitexin has shown anticancer effects in the cancer cell line by inducing apoptosis in several studies (Ninfali et al., 2017; He et al., 2016).

The effective concentration of each derivative of vitexin with molecular target and mechanism in different cancers has been summarized by Ninfali et al. (2017). For example, vitexin‐2‐O‐xyloside has a dose‐response anticancer effect (IC_50_ of 8.8 ± 0.8 μM, at 72 hr) and activated intrinsic pathway of apoptosis in T24 bladder cancer cells (Scarpa et al., 2016). An interesting issue is that vitexin had no toxicity against normal human bronchial epithelial 16HBE cells. Meanwhile, vitexin (40 μM) induced apoptosis possibly by suppressing PI3K/Akt/mTOR signaling in human nonsmall cell lung cancer A549 cells (NSCLC). Vitexin (2 mg/kg, i.p., 4w) also inhibited NSCLC tumor growth, increased the expression levels of Bax and cleaved caspase‐3, and decreased the expression of Bcl‐2 in the tumor tissue of mice (Liu, Jiang, Liu, & Luo, 2019).

Similarly, vitexin (10–50 μM) induced ROS generation in a dose‐dependent manner, possibly via the activation of JNK, and increased the expression of autophagy marker proteins Beclin‐1, Atg5, and microtubule‐associated protein light chain 3‐II (LC3‐II), which promote autophagy induction in colorectal carcinoma cells. Vitexin (25, 50, and 100 mg/kg, p.o.) inhibited the growth of colorectal carcinoma in mice xenograft model with low toxicity. It decreased in HSF‐1 (Heat shock transcription factor‐1) levels and increased in p‐JNK, LC3‐II, and ApoL1 levels (Bhardwaj et al., 2017).

Vitexin (100, 200 μg/ml, IC_50_ = 147 μg/ml) as an active constituent of *P. cineraria* had dose and time‐dependent anti‐proliferative activity in chronic myeloid leukemia (K‐562) cell line by inducing apoptosis through reducing SOD activity and elevating ROS, NO, and MDA (Sarkara, Mahapatrab, & Vadivel, 2020). Vitexin (10, 20 μM, 24 hr) suppressed the activation of NF‐κB and its key regulators (p65, IκBα and IKKs) and resulted in induction of apoptosis and inhibition of cell growth in nasopharyngeal carcinoma (NPC). In addition, vitexin (30 mg/kg, p.o., 2 weeks) decreased tumor growth through reducing of p‐p65 and Cyclin D1 expression in NPC xenograft mouse model (Wang, Cheng, Gu, & Yin, 2019).

Moreover, vitexin (10, 25, and 50 μM) dose‐dependently decreased ROS, upregulated Hsp 90, antioxidant enzymes (SOD, GR, and catalase), and MAPKs, and downregulated caspase‐3 and caspase‐4 in endoplasmic reticulum (ER) stress‐mediated autophagy in A549 cells. It therefore has cytoprotective and antiapoptotic effects (Bhardwaj, Paul, Jakhar, & Kang, 2015).

Three parameters of dose response, time of exposure to vitexin, and type of cancer cell lines are important for determining the antiapoptotic, apoptotic or proapoptotic effects of vitexin in cancer studies. It seems, however, that several factors are involved in directing the type of activity of vitexin in the cells, as previously noted. An important question is whether the target of vitexin is different in cancer cells compared to in normal cells. In other words, how can vitexin be used to activate apoptosis or autophagic cell death in cancer cells. Further studies can help answer these questions.

On the other hand, cooperation of vitexin (75 mg/kg, i.p., 21 days) with hyperbaric oxygen (HBO) therapy in glioma mouse model could sensitize the glioma radiotherapy by reducing glutathione peroxidase activity and glutathione content as well as expressions of HIF‐1α and VEGF in tumor tissues in SU3‐inoculated nude mice (Xie et al., 2019).

## 5 CONCLUSION

Vitexin is found in food sources and is used as an active component with herbal supplement. The present review study summarized all the protective effects of vitexin as an antioxidant against ROS, lipid peroxidation, and other oxidative damages with changes in oxidative and defense biomarkers in the nervous system, heart, and respiratory systems with possible mechanism on molecular and cellular signaling. Any activation (AMPK, Nrf‐2, and mTOR) or inhibition (JNK and BACE1) of the signaling pathways that depend on the antioxidant activity of vitexin in noncancer cells was also described. The diversity of the mechanisms of effect of vitexin against different oxidative stress models is the one of the most important points to consider regarding vitexin. Clinical studies are needed to further examine the protective effects of vitexin against oxidative stress‐related diseases, and as formerly noted, nanoparticles of it have been developed for increasing the bioavailability of vitexin.

## CONFLICT OF INTEREST

The authors declare that there is no conflict of interest.

## ETHICAL APPROVAL

The study did not involve any human or animal testing.
